# A cognitive perspective of an antibiotic timeout program

**DOI:** 10.1186/2047-2994-4-S1-P187

**Published:** 2015-06-16

**Authors:** M Jones, J Butler, C Graber, P Glassman, MH Samore, C Weir, MB Goetz

**Affiliations:** 1 Epidemiology, VA Salt Lake City HCS, Salt Lake City, USA; 2 IDEAS Center, VA Salt Lake City HCS, Salt Lake City, USA; 3 VA Greater Los Angeles HCS, Los Angeles, USA

## Introduction

Many antibiotic stewardship interventions are not well-enough grounded in theory to understand why they succeed or fail.

## Objectives

To understand impressions and antibiotic decision processes during a hospital’s transition to an informatics-supported antibiotic stewardship program that was based on Dual Process theories.

## Methods

A multi-pronged informatics intervention for antibiotic stewardship was implemented in a large US Veterans Affairs Medical Center. The objective of the program was to encourage providers to discontinue unnecessary antibiotics on or after antibiotic day three. It required the use of a template note for self-approval of antibiotics to nudge teams away from continuing vancomycin and piperacillin/tazobactam. It also included a paper handout on antibiotic day three to facilitate the cognitive process of determining whether antibiotics were still needed. During early intervention, six focus groups were conducted with users or potential users. Interviews were recorded and transcribed. Iterative thematic analysis was used to identify recurrent themes from feedback.

## Results

Themes emerged around alerting and orienting (“it reminds us to think about it”), deliberative reasoning (“it makes you think twice”; “You can already tell he’s getting better just by looking at all those graphs, they’re all turning the right way”), post-hoc justification and rationalization (“my clinical concern is high enough I think they need more aggressive therapy...”), impact on role responsibilities and relationships (“the template… forces the team to really discuss it”), and change management and impact on workflow processes (“I really don’t want to…go through [the approval template] so I just DC’d it without even trying”).

## Conclusion

Requiring a simple template nudged providers to think about whether they needed antibiotics. Thinking about antibiotics was further aided by clinical information. Nudges can be used to make it harder to continue undesired behaviors and easier to move to desired behaviors without seriously curtailing autonomy. When designing informatics interventions, a knowledge of clinician time, workflow, and thought processes could result in the design of effective nudges.

## Disclosure of interest

None declared.

